# Supplementary material for: Whole Genome Sequence Analysis of Cupriavidus necator C39, a Multiple Heavy Metal(loid) and Antibiotic Resistant Bacterium Isolated from a Gold/Copper Mine
Source: Microorganisms. 2023 Jun 7;11(6):1518. doi: 10.3390/microorganisms11061518 (PMC10304115; doi:10.3390/microorganisms11061518)
Supplement: Supplementary file 1 [file microorganisms-11-01518-s001.zip › microorganisms-2391139-supplementary.pdf]

Table S1 Primers used in this study

Primer pairs	Primer sequences	Usage
qB-F/qB-R	5' AAGGCCACGAGCGAATCAGA 3'<br>5' CATGACGCCCGCCTTGACGA 3'	For RT-qPCR of *phnB*
qI-F/qI-R	5' ACCGATCCACAGGGCATTGCAT 3'<br>5' CCTTCGCCGGACGTCGCTTC 3'	For RT-qPCR of *arsI*
qS-F/qS-R	5' ACACGACCTGAACACCGAAGCC 3'<br>5' ACACGACCTGAACACCGAAGCC 3'	For RT-qPCR of glutathione S-transferase gene *GST*
q39S-F/q39S-R	5' GTAGTCCACGCCCTAAACGA 3'<br>5' TAATCTTGCGACCGTACTCCC 3'	For RT-qPCR of 16S rRNA gene

Table S2 Antibiotic resistance genes (ARGs) of *C. necator* C39

Locus_tag	Identity	E_value	Subject_id	Antibiotic_Resistance	Description
JJQ59_00365	67.98	1.00E-175	ardb_543	fosmidomycin	Efflux pump/potassium antiporter system. RosA: Major facilitator superfamily transporter. RosB: Potassium antiporter.
JJQ59_02260	40.28	3.00E-49	ardb_2322	bacitracin	ABC transporter system, bacitracin efflux pump.
JJQ59_02735	51.08	9.00E-94	ardb_1750	kasugamycin	Specifically dimethylates two adjacent adenosines in the loop of a conserved hairpin near the 3'-end of 16S rRNA in the 30S particle. Its inactivation leads to kasugamycin resistance.
JJQ59_03110	42.61	4.00E-95	ardb_269	chloramphenicol	Resistance-nodulation-cell division transporter system. Multidrug resistance efflux pump.
JJQ59_03845	41.9	4.00E-15	ardb_1267	na_antimicrobials	Small Multidrug Resistance (SMR) protein family. Multidrug resistance efflux pump, which consists of two proteins.
JJQ59_08555	67.97	0	ardb_2089	aminoglycoside,tigecycline,fluoroquinolone,beta_lactam, tetracycline	Resistance-nodulation-cell division transporter system. Multidrug resistance efflux pump.
JJQ59_08560	94.1	0	ardb_2491	aminoglycoside,tigecycline,fluoroquinolone,beta_lactam, tetracycline	Resistance-nodulation-cell division transporter system. Multidrug resistance efflux pump.

JJQ59_08565	71.13	0	ardb_1137	aminoglycoside,tigecycline,fluoroquinolone,beta_lactam, tetracycline	Resistance-nodulation-cell division transporter system. Multidrug resistance efflux pump.
JJQ59_09360	41.75	1.00E-19	ardb_1205	na_antimicrobials	Small Multidrug Resistance (SMR) protein family. Multidrug resistance efflux pump, which consists of two proteins.
JJQ59_12630	63.3	1.00E-179	ardb_616	fosmidomycin	Efflux pump/potassium antiporter system. RosA: Major facilitator superfamily transporter. RosB: Potassium antiporter.
JJQ59_13300	51.82	1.00E-31	ardb_1789	aminoglycoside	Multidrug resistance efflux pump.
JJQ59_13435	44.51	2.00E-47	ardb_13	trimethoprim	Group A drug-insensitive dihydrofolate reductase, which can not be inhibited by trimethoprim.
JJQ59_14445	98.29	0	ardb_343	bacitracin	Undecaprenyl pyrophosphate phosphatase, which consists in the sequestration of Undecaprenyl pyrophosphate.
JJQ59_16570	45.89	2.00E-168	ardb_2181	penicillin	The enzyme has a penicillin-insensitive transglycosylase N-terminal domain (formation of linear glycan strands) and a penicillin-sensitive transpeptidase C-terminal domain (cross-linking of the peptide subunits)
JJQ59_16965	45.18	0	ardb_1593	chloramphenicol,fluoroquinolone	Resistance-nodulation-cell division transporter system. Multidrug resistance efflux pump.

JJQ59_17315	41.74	7.00E-149	ardb_2143	penicillin	The enzyme has a penicillin-insensitive transglycosylase N-terminal domain (formation of linear glycan strands) and a penicillin-sensitive transpeptidase C-terminal domain (cross-linking of the peptide subunits)
JJQ59_18140	47.27	0	ardb_188	aminoglycoside,glycylcycline	Resistance-nodulation-cell division transporter system. Multidrug resistance efflux pump.
JJQ59_18810	51.22	1.00E-121	ardb_1693	aminoglycoside,glycylcycline,macrolide,beta_lactam, acriflavin	Resistance-nodulation-cell division transporter system. Multidrug resistance efflux pump.
JJQ59_18815	61.59	0	ardb_1927	aminoglycoside,glycylcycline,macrolide,beta_lactam, acriflavin	Resistance-nodulation-cell division transporter system. Multidrug resistance efflux pump.
JJQ59_18820	48.95	1.00E-153	ardb_1138	aminoglycoside,tigecycline,fluoroquinolone,beta_lactam, tetracycline	Resistance-nodulation-cell division transporter system. Multidrug resistance efflux pump.
JJQ59_19940	48.28	2.00E-95	ardb_78	chloramphenicol	Resistance-nodulation-cell division transporter system. Multidrug resistance efflux pump.
JJQ59_19945	66.83	0	ardb_1646	chloramphenicol	Resistance-nodulation-cell division transporter system. Multidrug resistance efflux pump.
JJQ59_21875	51.88	1.00E-157	ardb_1137	aminoglycoside,tigecycline,fluoroquinolone,beta_lactam, tetracycline	Resistance-nodulation-cell division transporter system. Multidrug resistance efflux pump.

JJQ59_23635	60.38	3.00E-146	ardb_73	chloramphenicol	Resistance-nodulation-cell division transporter system. Multidrug resistance efflux pump.
JJQ59_23640	77.69	0	ardb_1645	chloramphenicol	Resistance-nodulation-cell division transporter system. Multidrug resistance efflux pump.
JJQ59_23645	53.96	6.00E-157	ardb_273	chloramphenicol	Resistance-nodulation-cell division transporter system. Multidrug resistance efflux pump.
JJQ59_24345	40.28	6.00E-50	ardb_2436	vancomycin	VanB type vancomycin resistance operon genes, which can synthesize peptidoglycan with modified C-terminal D-Ala-D-Ala to D-alanine--D-lactate.
JJQ59_24625	56.59	2.00E-162	ardb_2169	cephalosporin	Class C beta-lactamase. This enzyme breaks the beta-lactam antibiotic ring open and deactivates the molecule's antibacterial properites.
JJQ59_24830	55.52	2.00E-115	ardb_2089	aminoglycoside,tigecycline,fluoroquinolone,beta_lactam, tetracycline	Resistance-nodulation-cell division transporter system. Multidrug resistance efflux pump.
JJQ59_24835	62.82	0	ardb_2440	aminoglycoside,tigecycline,fluoroquinolone,beta_lactam, tetracycline	Resistance-nodulation-cell division transporter system. Multidrug resistance efflux pump.
JJQ59_24840	53.62	3.00E-153	ardb_1137	aminoglycoside,tigecycline,fluoroquinolone,beta_lactam, tetracycline	Resistance-nodulation-cell division transporter system. Multidrug resistance efflux pump.

JJQ59_25955	40.08	7.00E-56	ardb_1345	cloxacillin,penicillin	Class D beta-lactamase. This enzyme breaks the beta-lactam antibiotic ring open and deactivates the molecule's antibacterial properites.
JJQ59_26645	41.94	1.00E-74	ardb_737	polymyxin	Bifunctional enzyme that catalyzes the oxidative decarboxylation of UDP-glucuronic acid (UDP-GlcUA) to UDP-4-keto-arabinose (UDP-Ara4O) and the addition of a formyl group to UDP-4-amino-4-deoxy-L-arabinose (UDP-L-Ara4N) to form UDP-L-4-formamido-arabinose (UDP-L-Ara4FN). The modified arabinose is attached to lipid A and is required for resistance to polymyxin and cationic antimicrobial peptides.
JJQ59_26650	56.76	9.00E-138	ardb_736	polymyxin	Bifunctional enzyme that catalyzes the oxidative decarboxylation of UDP-glucuronic acid (UDP-GlcUA) to UDP-4-keto-arabinose (UDP-Ara4O) and the addition of a formyl group to UDP-4-amino-4-deoxy-L-arabinose (UDP-L-Ara4N) to form UDP-L-4-formamido-arabinose (UDP-L-Ara4FN). The modified arabinose is attached to lipid A and is required for resistance to polymyxin and cationic

					antimicrobial peptides.
JJQ59_37065	42.5	3.00E-26	ardb_1551	vancomycin	VanG type vancomycin resistance operon genes, which can synthesize peptidoglycan with modified C-terminal D-Ala-D-Ala to D-alanine--D-serine.
JJQ59_38205	48.83	2.00E-135	ardb_1137	aminoglycoside,tigecycline,fluoroquinolone,beta_lactam, tetracycline	Resistance-nodulation-cell division transporter system. Multidrug resistance efflux pump.
